# The effect of silver oxidation on the photocatalytic activity of Ag/ZnO hybrid plasmonic/metal-oxide nanostructures under visible light and in the dark

**DOI:** 10.1038/s41598-019-48075-7

**Published:** 2019-08-14

**Authors:** Azin Ziashahabi, Mirko Prato, Zhiya Dang, Reza Poursalehi, Naimeh Naseri

**Affiliations:** 1 Department of Materials Engineering, Tarbiat Modares University, Tehran, 14115-143 Iran; 2 Materials Characterization Facility, Istituto Italiano di Tecnologia (IIT), Via Morego 30, 16163 Genova, Italy; 3 Department of Nanochemistry, Istituto Italiano di Tecnologia (IIT), via Morego 30, 16163 Genova, Italy; 4 Department of Physics, Sharif University of Technology, P.O. Box 11555-9161, Tehran, Iran; 5 Condense Mater National Lab, Institute for Research in Fundamental Sciences (IPM), Tehran, Iran

**Keywords:** Photocatalysis, Nanoparticles

## Abstract

A new synergetic hybrid Ag/ZnO nanostructure was fabricated which is able to cause photocatalytic degradation (in high yields) of methylene blue under visible light as well as in the dark. In this nanostructure, ZnO was synthesized using the arc discharge method in water and was coupled with Ag via a chemical reduction method. X-ray photoelectron spectroscopy (XPS) and photoluminescence spectroscopy results confirmed the existence of defects in ZnO in the hybrid nanostructures; these defects act as electron traps and inhibit the recombination of electron-hole pairs. The absorption edge of the hybrid nanostructure shifts toward the visible region of the spectrum due to a combination of the Ag plasmonic effect and the defects in ZnO. Band-edge tuning causes effective visible light absorption and enhances the dye degradation efficiency of Ag/ZnO nanostructures. Silver oxidation in the hetero-structure changed the metal-semiconductor interface and suppressed the plasmonic enhancement. Nevertheless, the synthesized Ag/ZnO decomposed methylene blue in visible light, and the silver oxidation only affected the catalytic activity in the dark. This work provides insight into the design of a new and durable plasmonic-metal oxide nanocomposite with efficient dye degradation even without light illumination.

## Introduction

Organic dyes from industries such as textiles, paper, leather, printing inks and cosmetics release non bio-degradable contaminants into water. Photocatalysis is a promising route to remove toxic dyes from waste water. In this regard, semiconductor photocatalysts have attracted the attention for water treatment and environmental remediation applications. In comparison to conventional wastewater treatment methods, applying nanostructured semiconductors is an efficient and economically favorable technique.

In particular, ZnO has been extensively used due to its non-toxicity, low cost and high stability against photo-corrosion in photo degradation studies^1,2^. ZnO can completely remove and degrade organic dyes such as methylene blue (MB) to CO_2_ and H_2_O under UV irradiation. However, the main drawback of ZnO in photocatalytic applications is the wide band gap value of 3.3 eV, which requires UV illumination. This large band gap suppresses the solar driven photocatalysis reactions. Therefore, different modifications such as metal and non-metal doping, dye sensitization, and coupling with other semiconductors have been attempted to enhance the visible light absorption of ZnO. Coupling with noble metals is an effective approach to enhance the visible light absorption and photocatalytic activity of ZnO. Plasmonic nanoparticles have a strong absorption in the visible region of the spectrum due to localized surface plasmon resonances (LSPR). These nanoparticles are able to strongly concentrate the incident light on the surface and enhance photochemical reactions. When these plasmonic nanoparticles attach to semiconductors, a Schottky barrier will form at the interface between the metal and the semiconductor. The formation of this metal-semiconductor hetero-junction is an effective way to enhance charge carrier separation and improve the photocatalytic efficiency^3–5^. In this regard, decoration of TiO_2_/ZnO with noble metal nanoparticles is a promising method to reduce the recombination of the photogenerated electrons and enhance the photo-degradation activity^6–10^. However, size, shape, inter-particle coupling and the surrounding medium of the plasmonic nanostructure affect the solar absorption and overall photocatalytic efficiency of the hybrid hetero-structures. Therefore, to tune the optical properties in hybrid plasmonic nanostructures, these parameters need to be accurately adjusted. Bhattacharyya *et al*. modified the band gap of TiO_2_ nanotubes by controlling the size/morphology and Ag/Au loading^11^. Kumar and Sarswat investigated the surface plasmon polaritons interactions with nanomaterials^12^.

Different ZnO-noble metal plasmonic nanohybrids have been synthesized for photocatalytic dye degradation^13–18^. It is believed that the combination of surface defects in ZnO together with the presence of Ag nanoparticles leads to the visible light photocatalytic activity of Ag/ZnO hybrid nanostructure (HNS)^19–21^. Defects introduce states below the conduction band minimum of ZnO, and these states act as electron traps, inhibiting electron recombination and finally enhancing both carrier separation and photocatalytic activity^20,21^. In this regard, Ag/ZnO heterostructures have been extensively used to enhance the photo-degradation of different organic dyes under UV and visible light^15,19,22–25^. On the other hand, the dye degradation activity of such heterostructures has not yet been addressed in the absence of light illumination. This research sheds light to dye degradation mechanism of Ag/ZnO HNS under visible light and also in the dark.

We describe here a two-step method to prepare a synergetic Ag/ZnO hybrid nanostructure for complete degradation of MB. The as-prepared hybrid nanostructure shows highly efficient photocatalytic activity for degradation of MB under visible light and even in the dark. The pristine ZnO nanoparticles were prepared via arc discharge of zinc rods in water and exhibit visible light degradation. The synthesized ZnO nanopowder are then hybridized with Ag nanoparticles following a wet chemical method. Furthermore, we investigated the effect of silver oxidation on Ag-ZnO interface properties and on the degradation activity. A systematic characterization was done to study the morphology, phase, composition, and photocatalytic properties of the nanostructures. The defect states and possible charge transfers at the metal/semiconductor interface were investigated using photoluminescence spectroscopy. The surface chemical composition and the presence of defects in the nanostructures, which lead to visible light photocatalytic activity, were confirmed by XPS. The crystallinity and phase composition of the nanostructures were studied with X-ray diffraction (XRD) analysis. The microstructure, morphology and quality of the silver dispersion in Ag/ZnO HNS were analyzed with transmission electron microscopy (TEM), HRTEM, high resolution scanning electron microscopy (HR-SEM) and energy dispersive X-ray spectroscopy (EDX). Finally, UV-vis spectroscopy was used to study the optical absorption of the nanostructures. Although ZnO is not usually photocatalytically active in the visible light, we have demonstrated a facile arc discharge method to synthesize visible light active ZnO nanoparticles. In addition, coupling ZnO nanoparticles with Ag further enhances dye degradation efficiency both in the dark and under solar illumination. These results could be useful with regard to developing a nanostructure with high dye degradation properties even in the dark.

## Results and Discussion

### Morphology and chemical composition characterization

**Figure 1:**
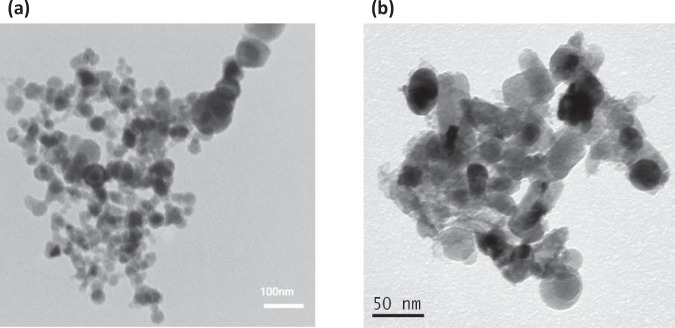
TEM image of (**a**) pristine ZnO and (**b**) the as-prepared hybrid Ag/ZnO nanostructure.

**Figure 2:**
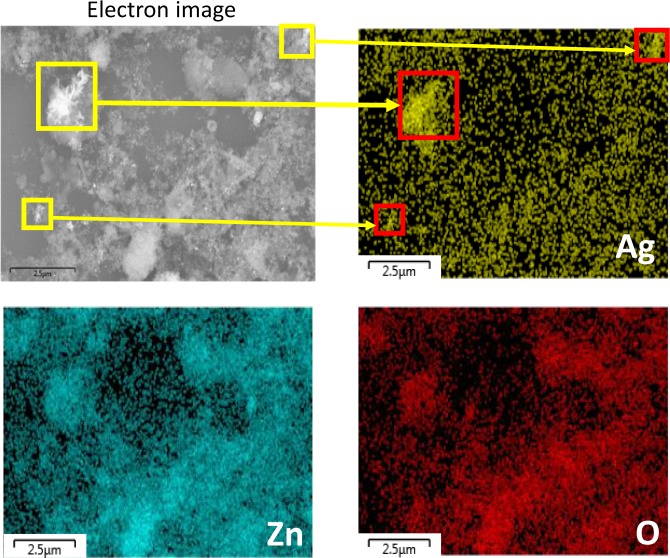
HR-SEM image and EDX maps of Ag, Zn and O atoms in fresh Ag/ZnO HNS. Yellow and red squares show high Ag-concentrated regions.

**Figure 3:**
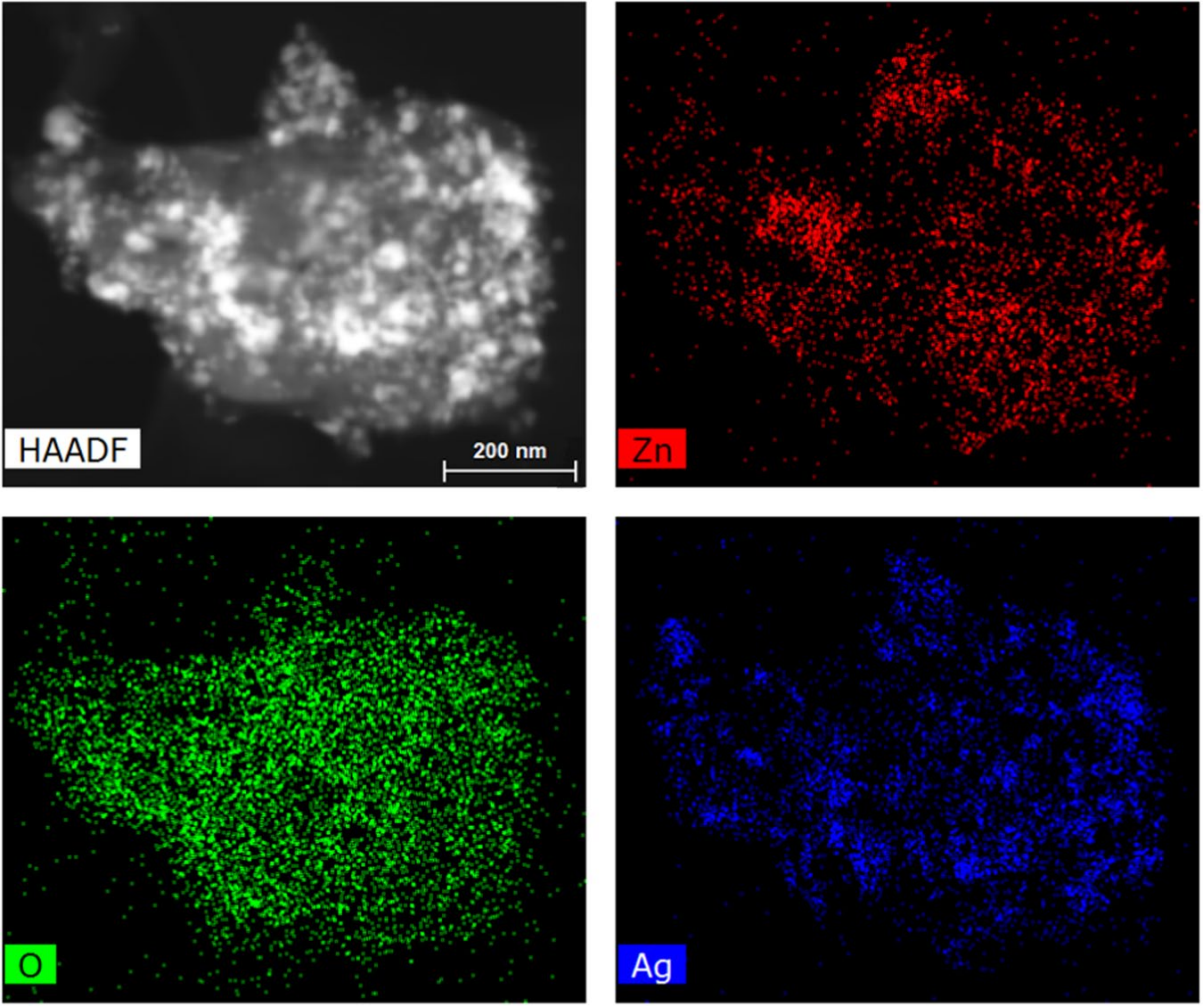
STEM-HAADF image and the EDX maps of Ag, Zn and O atoms in aged Ag/ZnO HNS.

**Figure 4:**
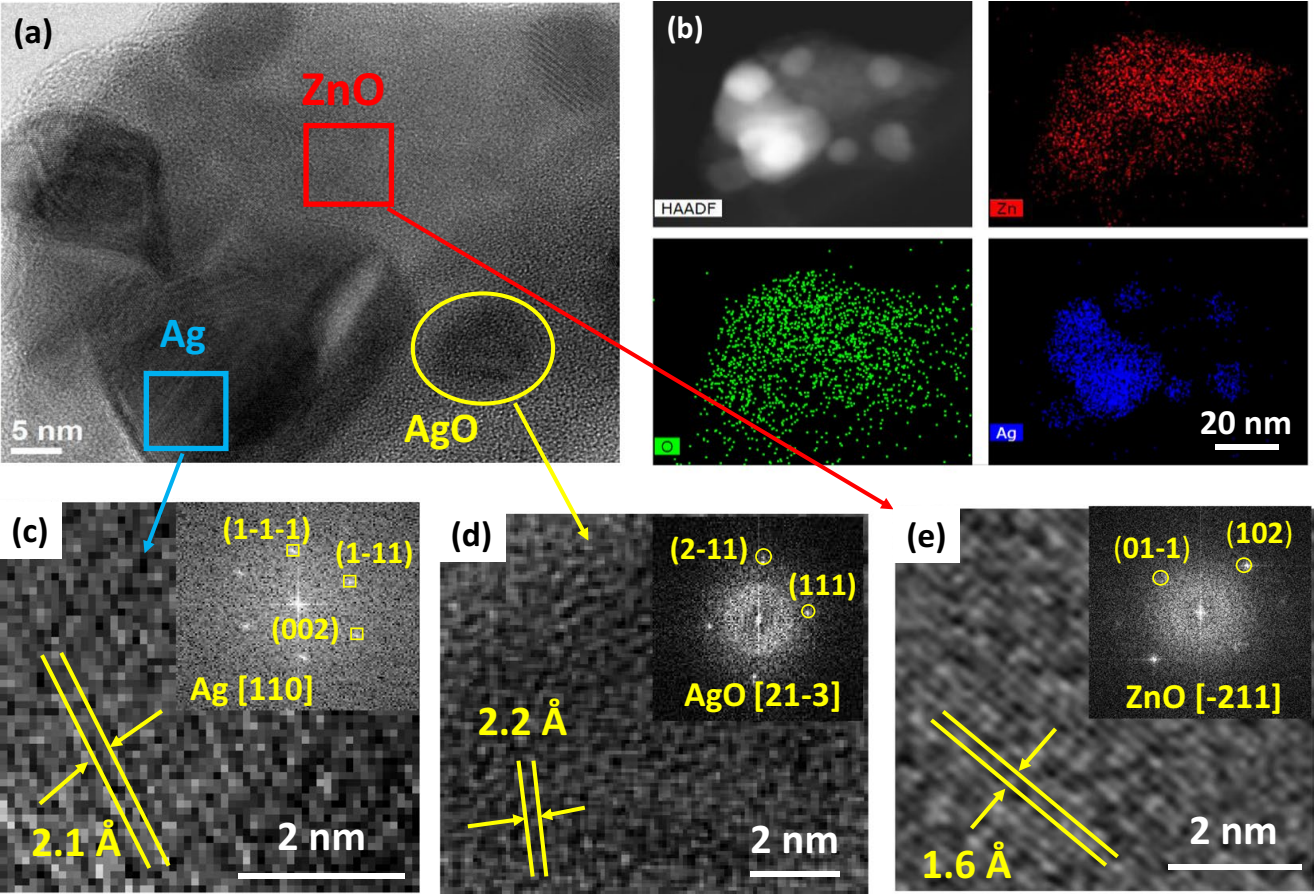
(**a**) HRTEM image of aged Ag/ZnO HNS, (**b**) corresponding STEM-HAADF image and the Zn, O and Ag elemental maps obtained from EDX mapping, HRTEM images showing lattice fingers of (**c**) silver, (**d**) silver oxide and (**e**) zinc oxide (the insets are the FFT images of the selected areas).

TEM images of ZnO and the as-synthesized Ag/ZnO HNS are shown in Fig. 1. Figure 1a illustrates pristine ZnO nanoparticles with spherical shape and Fig. 1b depicts aggregation of nanoparticles with addition of Ag in fresh hybrid sample. To study the chemical composition and distribution of Ag in the hybrid nanostructure, EDX analysis was performed. Figure 2 illustrates an HR-SEM image of the as-synthesized Ag/ZnO HNS and its corresponding EDX maps. The EDX maps clearly show very similar distribution of Zn and O atoms while the Ag atoms distribution is different. The areas that are enclosed by yellow and red boxes in Fig. 2, show the most Ag-concentrated regions in fresh Ag/ZnO HNS. In this research, aged Ag/ZnO HNS refers to the sample after being applied in photocatalytic degradation process, which there is AgO phase. A full discussion about different crystallographic phases in hybrid nanostructures is in the XRD section. Figure 3 shows high angular annular dark field (HAADF)-scanning TEM (STEM) image of aged Ag/ZnO HNS and the corresponding elemental mapping. Figure 3 shows very similar distribution of Zn and Ag atoms in the aged sample while the O atoms distributed almost everywhere. To investigate oxidation of silver atoms and the atomic structure in the aged Ag/ZnO HNS the high resolution TEM (HRTEM) analysis was performed. Figure 4a shows the HRTEM of an aged Ag/ZnO HNS, which shows silver and silver oxide nanoparticles decorating the ZnO nanostructure. Figure 4b is the HAADF-STEM image of Fig. 4a with the corresponding EDX mapping of Zn, O and Ag atoms. Figure 4c,d,e shows the magnified view of the three regions in Fig. 4 a, showing lattice fringes and corresponding fast Fourier transform (FFT). The atomic structures of the three regions match with Ag, AgO and ZnO respectively, in which the lattice spacing of 2.1 Å, 2.2 Å and 1.6 Å of the labelled fringes correspond to the (1–1 1), (111) and (102) interplanar spacing of Ag, AgO and ZnO respectively (according to ICSD 44387, 27659, and 26170 respectively for Ag, AgO, and ZnO).

### XRD characterization

**Figure 5:**
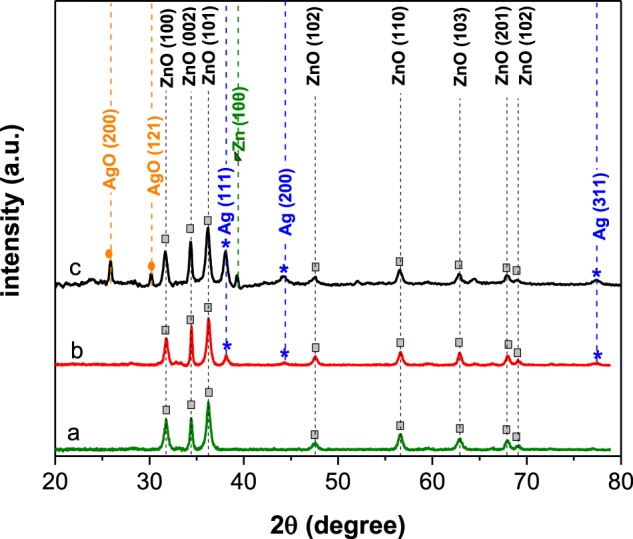
XRD patterns of (**a**) pristine ZnO, (**b**) Ag/ZnO fresh and (**c**) Ag/ZnO aged sample nanoparticles.

The crystallinity, phase and composition of the nanoparticles were determined with XRD analysis. Figure 5 shows the XRD patterns of pure ZnO, the as-synthesized, and aged Ag/ZnO HNS for 2θ from 20 to 80 degrees. Eight diffraction peaks of wurtzite ZnO were observed and indexed in Fig. 5. These peaks are in both pristine ZnO and in Ag/ZnO hybrid nanostructures without any shifts, thus suggesting that the hybridization with Ag nano-colloids did not alter the crystal structure of the ZnO domains. In Fig. 5b, the XRD pattern of a fresh Ag/ZnO sample shows two extra peaks at 38.1° and 44.2°and one tiny peak at 77.4°, corresponding to the face centered cubic (fcc) crystalline structure of the silver (111), (200) and (311) planes respectively. No peaks belonging to silver oxide phases were observed. The ZnO and Ag peaks were ascribed to the ICDD PDF-2 entries 01-076-0704^26^ and 01-087-0717^27^ respectively. In aged Ag/ZnO HNS, in fact, two other phases were observed in addition to the metallic silver and wurtzite ZnO ones. In Fig. 5c, there are two peaks at 25.8° and 30.1° (corresponding to the AgO) and one peak at 39.3° (which is related to the metallic Zn) according to the ICDD PDF-2^28^ entries 01-084-1108 and 00-001-1238^29^ respectively for the AgO and Zn peaks. It should be noted that AgO is a mixture of Ag_2_O and Ag_2_O_3_, and it is difficult to distinguish which one really exists in the aged Ag/ZnO HNS. However, Ag_2_O is unstable under light irradiation, and it decomposed to AgO and Ag^30^. The Ag/ZnO HNS is oxidized in aqueous solution during degradation process and illumination fastens this reaction. The Zn peak which is observed in the aged Ag/ZnO sample is due to remaining Zn nanoparticles from the precursor.

### XPS analysis

**Figure 6:**
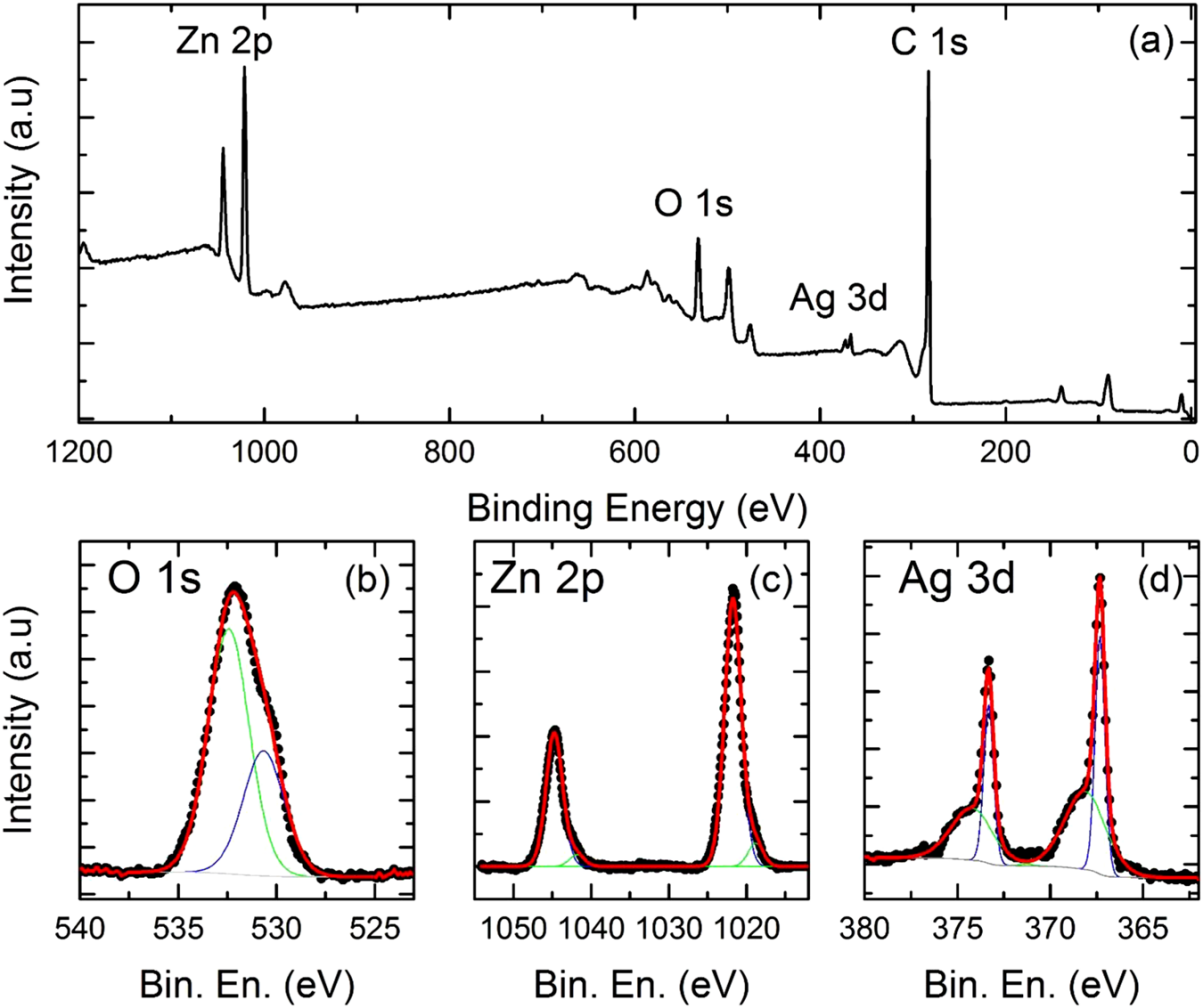
(**a**) Complete XPS spectrum of an Ag/ZnO nanostructure, high-resolution spectra of the (**b**) O 1 s, (**c**) Zn 2p and (**d**) Ag 3d orbitals.

XPS measurements were used to identify the elemental composition and surface chemical state of the Ag/ZnO HNS. The peak positions were calibrated based on the carbon C 1 s peak, centered at binding energy of 284.8 eV. In Fig. 6a, a wide XPS spectrum displays the Zn 2p, O 1 s, Ag 3d and C 1 s peaks. No other elements were identified. All experimental data in the high-resolution XPS spectra were deconvoluted and fitted with Voigt profiles. Figure 6b reveals that the O 1 s peak in Ag/ZnO HNS is composed of two peaks at 530.6 and 532.6 eV. The peak with a lower binding energy is attributed to the O^2−^ lattice oxygen in the ZnO wurtzite crystal structure^31^. The peak near 532.6 eV is ascribed to the adsorbed oxygen species. This peak may be related to the adsorbed H_2_O, ^−^OH, O^−^, O^2−^, -CO ions that are on the surface^21,32–38^. In Fig. 6c, two characteristic peaks of Zn 2p_1/2_ and 2p_3/2_ are observed at 1044.7 eV and 1021.6 eV respectively. These two peaks are related to Zn^2+^ due to the presence of Zn-O bonds^39^. These asymmetric peaks have shoulders in lower binding energies, and they are located at 1042.1 eV and 1019.0 eV. The shoulders with lower binding energies indicate that Zn is in a lower oxidation state than in ZnO. In Fig. 6d, the spectrum collected on the energy region that is typical for Ag 3d peaks is reported. Two sets of peaks are clearly visible: one has Ag 3d_3/2_ and Ag 3d_5/2_ peaks at 374.3 eV and 368.2 eV, which are assigned to the presence of metallic Ag; the other has Ag 3d peaks at 373.3 eV and 367.3 eV, which are linked to the presence of AgO. These findings are in agreement with previously reported results^40^.

### Optical properties

**Figure 7:**
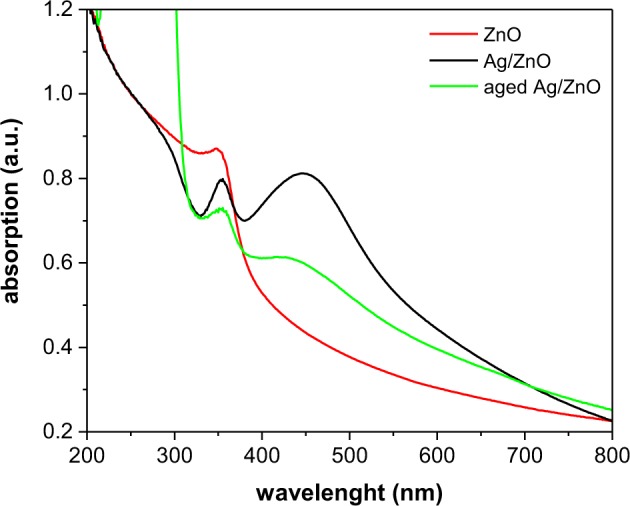
Optical absorption spectra of ZnO and hybrid Ag/ZnO nanoparticles.

To investigate the effects of silver coupling on the optical absorption and band edge energy of ZnO nanoparticles, UV-vis spectroscopy was performed. Figure 7 shows the absorption spectra of pure ZnO and Ag/ZnO HNS. ZnO shows a high absorption in UV region of the spectrum and a band-edge near 400 nm. It can be observed that the optical absorption increases significantly in the visible region of the spectrum by coupling the silver to the ZnO nanoparticles. The main feature of the optical absorption of Ag/ZnO HNS is a broad peak at 446 nm, which appears to be due to the LSPR of silver. The other peak, which is observed at a wavelength of 353 nm, is the exciton peak of ZnO nanoparticles^41,42^. In aged Ag/ZnO HNS, both plasmonic and excitonic peaks were damped. Furthermore, the plasmonic peak shifted to 428 nm due to the oxidation of the silver nanoparticles^43^. High visible light absorption and effective charge carrier separation is required for efficient dye degradation. If the photo-generated electrons recombine very quickly, no electron-hole pairs would remain to carry out photocatalytic reactions. A less effective dye degradation is predicted in the case of aged hybrid samples due to the fact that they absorb visible light less effectively than the fresh samples.

**Figure 8:**
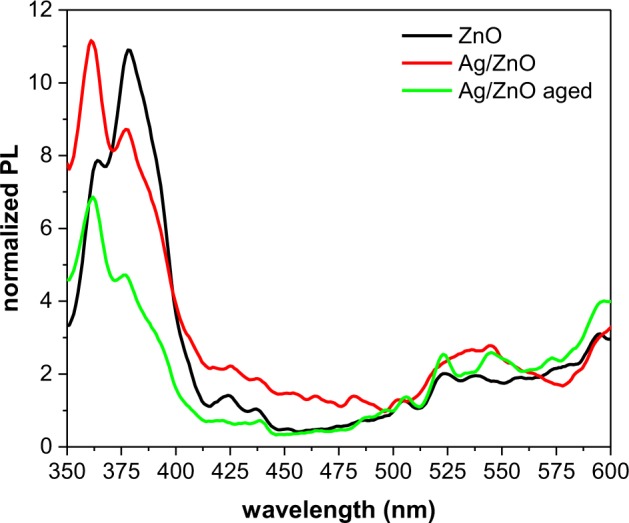
Normalized PL spectra of ZnO and Ag/ZnO hybrid nanoparticles.

To study the electronic structure of the nanoparticles, a PL analysis was carried out. The room temperature PL spectra were recorded with an excitation wavelength of 320 nm. Figure 8 illustrates the normalized PL spectra of the nanostructures. Normalizations were calculated according to the absorption spectra of different samples at 320 nm. ZnO and Ag/ZnO HNS both exhibit two distinct UV emissions. These two UV peaks appear as a result of near band edge emissions (NBE), but they are attributed to different effects in the nanostructures. The emission peak at 364 nm originates from a direct recombination of free excitons (FEs), while the emission peak at 378 nm is related to bound excitons (BEs)^44^. This recombination feature, which is caused by BEs, has been observed previously but its origin is still unknown^45–48^. It has been ascribed to different BEs from crystal defects such as donor BEs^49,50^, acceptor BEs^51^, and excitons bound to extended structural defects^46,52^. As Fig. 6 depicts, the dominant emission in ZnO is from the BEs, while the FEs show a more intense emission in the Ag/ZnO samples. This means that the Ag coupling, increased free exciton emissions and quenched the bound exciton emissions in the hybrid nanostructures. Other features of the PL analysis include several emissions in the visible region of the spectra from 500 nm to 600 nm. Figure 8 shows that there are several green and yellow emission peaks in both the ZnO and Ag/ZnO HNS at 505 nm, 523 nm, 545 nm, and 595 nm. These peaks are due to deep level emissions (DLE) from different crystal defects^53,54^. The reason for the green emission in ZnO is not yet clear; it is, in fact, a highly controversial topic. Some calculations suggest that oxygen vacancies cause the green emission^53,55^, while others conclude that it is due to oxygen antisite defects^56,57^, and a few reports ascribe it to a zinc vacancy^58^. It has been suggested that the yellow emission near 598 nm originates from oxygen interstitial defects^59,60^. In Fig. 8, the intensities of these visible emissions in hybrid Ag/ZnO are quite similar to that of pristine ZnO. Generally, in doped Ag/ZnO nanostructures, Ag ions may place in substitutional Zn^2+^ or in interstitial positions, and this creates more lattice defects. This leads to more electron-hole separations, and it quenches the visible emissions^61^. In this research, we did not observe any significant changes in visible emissions with Ag coupling. However, any change of the PL emissions in Ag/ZnO HNS depends on the band alignment and Schottcky barrier properties.

### Photocatalytic activity

**Figure 9:**
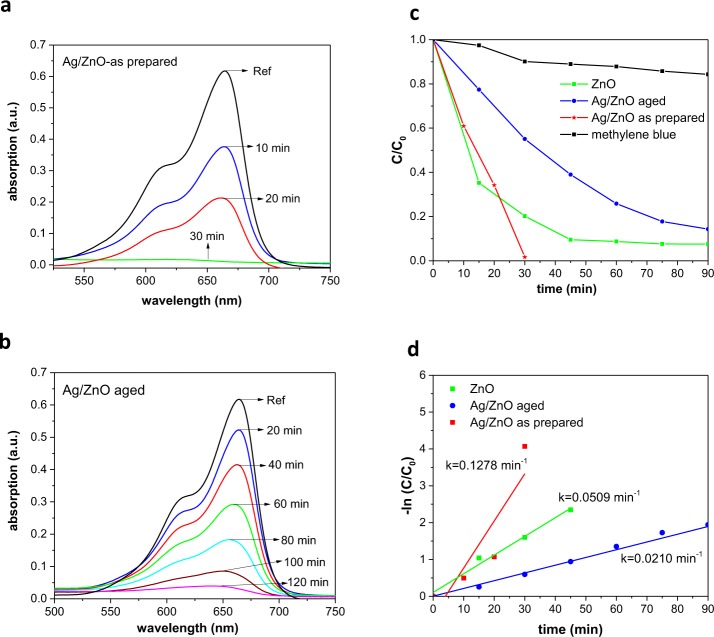
Methylene blue degradation pathway of (**a**) fresh Ag/ZnO HNS for 30 minutes without illumination in dark, (**b**) aged Ag/ZnO HNS under visible light illumination, (**c**) kinetics of photocatalytic degradation by different samples and (**d**) variation of −ln (C/C_0_) with irradiation time.

The photocatalytic activity of pure ZnO and Ag/ZnO HNS was evaluated by MB dye degradation in the dark and under solar irradiation. At low concentrations of the dye, the degradation kinetics of the MB can be described using the first-order Langmuir equation^62^:

1 $$\mathrm{ln}(\frac{C}{{C}_{0}})=-\,kt$$

in which t is the time of the reaction, C_0_ is the initial concentration of MB, C is the concentration at different times after exposure, and k is the first order rate constant of the degradation reaction. The photocatalytic activity and MB degradation kinetics for Ag/ZnO HNS is presented in Fig. 9. Figure 9a,b shows the absorption spectra of MB during degradation process by fresh and aged Ag/ZnO HNS respectively. It should be noticed that the degradation process by the fresh sample was carried out without light illumination and was completed after 30 minutes. For aged Ag/ZnO HNS the degradation was carried out under solar simulator and it took 120 minutes to remove the dye. Figure 9c shows C/C_0_ plot versus irradiation time for ZnO and Ag/ZnO HNS. From Fig. 9c it can be clearly seen that ZnO shows highly efficient visible light degradation that might be due to the presence of defects as well as to its oxygen rich surface. Coupling ZnO with Ag further enhanced the efficiency of the dye degradation in the dark, and this could be as a result of the electron trap states that were introduced by Ag. In addition, the combination of defective ZnO and LSPR of Ag nanoparticles makes the Ag/ZnO HNS an interesting candidate for dye degradation in visible light and dark. However, aged Ag/ZnO HNS shows less degradation efficiency due to oxidation of silver and less visible light absorption. Figure 9d shows the degradation reaction kinetics, the reaction rate constant was measured from the slopes of the −ln(C/C_0_) plots versus time. The calculated k values obtained 5.09 × 10^−2^, 12.78 × 10^−2^, and 2.10 × 10^−2^ min^−1^ for ZnO, fresh Ag/ZnO and aged Ag/ZnO respectively.

**Figure 10:**
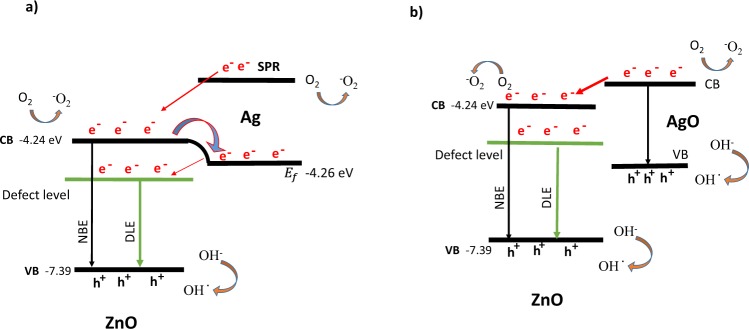
Schematic band diagram and possible electron transfers of (**a**) as prepared Ag/ZnO HNS, which formed a metal/semiconductor heterojunction, and (**b**) an aged Ag/ZnO HNS sample, which formed a p-n junction. Band level positions of ZnO, AgO and the Fermi level of Ag are taken from refs^63,64^.

The mechanism behind the photocatalytic degradation activity of hybrid Ag/ZnO nanostructures can be explained as follows. For the fresh sample, there are three possible electron transfers at the metal-semiconductor interface, which is explained schematically in Fig. 10a (the energy levels are taken from ref.^63^). The first one is a direct electron transfer from Ag to ZnO: the electrons in the conduction band of the Ag nanoparticles are excited to the SPR state, then the excited electrons will transfer to the ZnO conduction band. In second mechanism, the electrons in the ZnO conduction band may transfer to the Fermi state of the Ag nanoparticles. In the third mechanism, the electron transfer depends on the position of energy levels of different defects in ZnO, and the electrons in the Fermi state of the Ag nanoparticles may transfer to the defect levels of ZnO. Electron transfer through these mechanisms will inhibit the recombination of charge carrier in fresh Ag/ZnO HNS. However, the electron transfer in aged Ag/ZnO HNS is more complicated due to the oxidation of silver. In this case, there are several metal semiconductor interfaces between ZnO, Ag, AgO and Zn. From the XRD results, it was found that the aged Ag/ZnO HNS are mostly composed of ZnO and AgO. Therefore the ZnO/AgO semiconductor/semiconductor interface is likely the dominant one. Figure 10b shows the schematic band diagram of AgO/ZnO (the AgO energy levels were taken from ref.^64^). AgO is a p-type semiconductor with a 1.7 eV band gap energy^64,65^. When silver is oxidized in the HNS, an AgO/ZnO p-n nano heterojunction is formed, and the electrons will transfer from ZnO to AgO. In the p-n junction, the electrons transfer from an n-type ZnO region to a p-type AgO region in order to reach a thermal equilibrium. Therefore, ZnO is positively charged, while AgO has a negative charge, and an electric field is generated at the junction. As is shown in Fig. 10b, the excited electrons in the AgO conduction band may transfer to the ZnO conduction band, and holes can transfer to the AgO valence band due to the formation of an inner electric field at the p-n junction.

### Degradation mechanism

To investigate the exact mechanism of the MB dye removing in the dark and to make sure whether it is surface adsorption or degradation, series of experiments were carried out. In order to study desorption process of MB from the surface of the fresh Ag/ZnO, we used combination of thermal and chemical regeneration technique^66–69^. In this regeneration method, the sample is heated after the dye adsorption and the desorption process performed using ethanol or acetonitrile^70^. Typically, samples with high surface area exhibit a high adsorption capacity of the dye. In our case, the surface area of the samples obtained 32.3, 18.7, and 28.0 m^2^/g for ZnO, fresh Ag/ZnO, and aged Ag/ZnO (BET, N_2_) respectively. As the BET surface area values are not high we do not expect high adsorption capacity of the MB by the samples.

**Figure 11:**
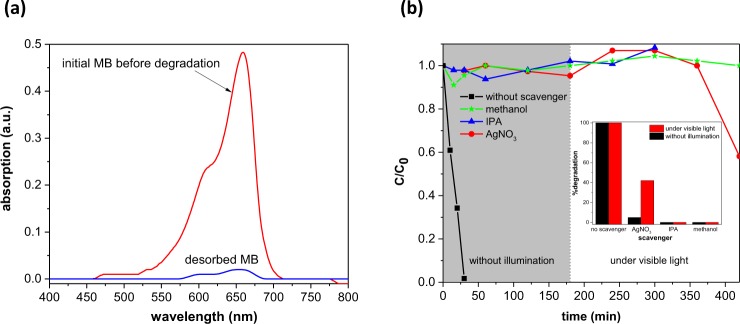
(**a**) Absorption spectra of the MB before degradation and the desorbed dye from the fresh Ag/ZnO sample (**b**) plot of C/C_0_ as a function of time for MB degradation in the presence of different scavengers performed without illumination and under visible light (the first 180 minutes was run in the dark). The inset shows the % degradation in the presence of different scavengers.

In the desorption experiment, after complete dye removing the photocatalyst was separated from the dye and heated at 160 °C for 1 hour. Then the powder  redispersed in ethanol and stirred for 2 hours. Every 15 minutes 1.5 ml of the solution collected, centrifuged and the supernatant used for the spectroscopy. The optical absorption spectrum of the desorbed MB did not change during 2 hours of the measurement. Figure 11 a shows the optical absorption spectra of the initial MB before adsorption and the desorbed dye from the sample. From Fig. 11 a, one can conclude that the concentration of the desorbed MB from the sample is very low which did not change after 2 hours. Hence, the ethanol contains very little amount of desorbed MB from the sample while Fig. 9 a shows that the MB was completely removed after 30 minutes without light illumination and the color of the nano-powder did not changed. Therefore, in this experiment adsorption should not be the dominant effect in the MB removing.

*In situ* capture experiments also carried out to evaluate the MB degradation under visible light and without illumination. Different scavengers were used to determine the active species generated during the degradation process. Isopropyl alcohol (IPA), methanol, and AgNO_3_ were used to capture the generated OH radicals, holes, and electrons during the reaction. Dye degradation experiment was performed in the presence of scavengers with 10 mM concentration. As shown in Fig. 11b, by using scavengers, degradation of the MB completely quenched in the dark. This result reveals that all the generated reactive species play an important role in the MB degradation activity and capturing each of them will eliminate the dye degradation. The use of hole and hydroxyl radical scavenger, also hindered the photocatalytic degradation under visible light. In case of AgNO_3_, as an electron scavenger under the visible light illumination, 40% of the dye was degraded and it is probably due to reducing of silver nitrate under illumination and loading some amount of metallic silver which may cause regeneration of surface electrons and increasing the degradation activity.

Performing these batch experiments, we can conclude that the dye degradation in the dark is due to generation of different active species on the surface of the fresh Ag/ZnO sample which lead to a high degradation efficiency.

## Conclusion

We synthesized ZnO nanoparticles using an arc discharge method in water, and successfully coupled them with Ag nanoparticles by means of a chemical reduction synthesis. XRD results revealed that fresh Ag/ZnO HNS were composed of wurtzite ZnO and metallic silver, but this composition changed as the sample aged. In addition to the ZnO and Ag peaks, a metallic Zn peak and two other peaks ascribed to silver oxide were also observed in the XRD pattern of aged Ag/ZnO HNS. Ag/ZnO HNS efficiently degraded the dye both under visible light irradiation and in the dark, without the involvement of illumination. Ag plasmonic effects, together with defects in the ZnO, lead to a high yield photocatalytic activity in degradation of MB. Under visible light irradiation, the enhanced degradation activity can be attributed to the higher visible light absorption of Ag/ZnO HNS. However, the defect levels in the ZnO and metallic Ag electron traps are responsible for the effective charge separation and photocatalytic activity in the dark. The present work provides a method for synthesis a plasmonic/metal-oxide nanostructure with efficient dye degradation in visible light and dark.

## Methods

### Synthesis of ZnO nanoparticles

Zinc rods (99.9%), trisodium citrate dehydrate (>98%) and silver nitrate (>99%) were purchased from sigma-aldrich. ZnO nanoparticles were prepared using a DC arc discharge method in water. The complete synthesis and oxidation process is discussed elsewhere^71^. Two zinc rods, which were used as electrodes, were immersed in 200 ml of deionized water. After applying a 100 A current to the electrodes, the electric discharge created a plasma in the short distance between the electrodes. The generated plasma caused the zinc electrodes to evaporate, creating ZnO nanoparticles. After complete oxidation in water, a white ZnO precipitate was collected. This precipitate was washed with ethanol and acetone several times before finally being dried at 40 °C for 3 hours.

### Synthesis of Ag/ZnO hybrid nanostructures

For the synthesis of Ag/ZnO HNS, colloidal Ag nanoparticles were prepared using a chemical reduction method. In this procedure, 36 mg of AgNO_3_ was dissolved in 200 ml of deionized water. This solution was then heated to 100 °C. Next, 4 ml of trisodium citrate 1 wt. % was added as a reducing agent, and the temperature of the solution was kept at 100 °C for one hour. The solution was then stirred and cooled down to room temperature, when it turned to a yellowish color. After preparing Ag colloidal nanoparticles, 20 mg of ZnO nanoparticles was dispersed in 20 ml of the prepared Ag colloid via a sonication process for 30 minutes. The solution was stirred for 24 hours at room temperature to obtain a brown precipitate. Finally, the precipitate was centrifuged, collected, and washed with ethanol and deionized water several times.

### Photocatalytic activity

The photocatalytic activity of the Ag/ZnO hybrid nanoparticles was determined by measuring the degradation of the MB dye under solar irradiation. 3 mg/L of an aqueous dye solution was prepared in deionized water, and this was used for all degradation measurements. In each test, 3 mg of the photocatalyst was added to 30 ml of the MB solution, and the solution was stirred in the dark for 60 minutes to reach an adsorption-desorption equilibrium. After keeping the solution in the dark for 60 minutes, irradiation was performed with visible light under a 100 W xenon lamp with a power density of 100 mW cm^−2^. Every 15 minutes, 3 ml of the solution was removed and centrifuged at 10000 rpm for 5 minutes, and UV-vis spectroscopy was recorded for the supernatant.

### Characterization

The crystalline structure of the samples was identified by XRD analysis using an Empyrean X-ray diffractometer equipped with a Cu K_α_ X-ray tube with a wavelength of 1.5406 Å. Morphology of nanoparticles was studied with TEM microscope, CM30, Philips at 150 kV. High resolution SEM-EDX analysis was done to characterize chemical composition and elemental distribution with high resolution SEM (JEOL JSM-7500FA). HRTEM and HAADF-STEM were performed on a JEOL JEM‐2200FS microscope equipped with a 200 kV field emission gun, a CEOS spherical aberration corrector for the objective lens and an in‐column image filter (Ω‐type). STEM-EDX elemental maps were obtained by a Bruker Quantax 400 system with a 60 mm^2^ XFlash 6 T silicon drift detector (SDD) equipped on this same microscope. UV-vis spectroscopy was carried out with a Varian Cary 300 spectrophotometer to measure the optical absorption spectra. Room temperature PL spectra were recorded by a Varian Cary Eclipse fluorescence spectrophotometer with an excitation wavelength of 320 nm. XPS analysis were performed with a Kratos Axis Ultra^DLD^ setup, using a monochromatic Al Kα source operated at 20 mA and 15 kV. Wide scans were acquired at a pass energy of 160 eV and with an energy step of 1 eV, while high resolution scans were acquired for the energy regions that are typical for Zn, O and Ag peaks at a pass energy of 20 eV and an energy step of 0.1 eV. Data were analyzed with CasaXPS software, version 2.3.17. Brunauer–Emmett– Teller (BET) surface area were determined using N_2_ adsorption isotherm measured by a Micromeritics Tristar II 3020 instrument.
